# Ionizing Radiation and Chronic Lymphocytic Leukemia

**DOI:** 10.1289/ehp.7433

**Published:** 2004-10-21

**Authors:** David B. Richardson, Steve Wing, Jane Schroeder, Inge Schmitz-Feuerhake, Wolfgang Hoffmann

**Affiliations:** ^1^Department of Epidemiology, School of Public Health, University of North Carolina at Chapel Hill, Chapel Hill, North Carolina, USA; ^2^Department of Physics (retired), University of Bremen, Germany; ^3^Institute for Community Medicine, Division of Health Care Epidemiology and Community Health, Ernst-Moritz-Arndt-University Greifswald, Greifswald, Germany

**Keywords:** chronic lymphocytic leukemia, compensation, ionizing radiation, radiogenicity

## Abstract

The U.S. government recently implemented rules for awarding compensation to individuals with cancer who were exposed to ionizing radiation while working in the nuclear weapons complex. Under these rules, chronic lymphocytic leukemia (CLL) is considered to be a nonradiogenic form of cancer. In other words, workers who develop CLL automatically have their compensation claim rejected because the compensation rules hold that the risk of radiation-induced CLL is zero. In this article we review molecular, clinical, and epidemiologic evidence regarding the radiogenicity of CLL. We note that current understanding of radiation-induced tumorigenesis and the etiology of lymphatic neoplasia provides a strong mechanistic basis for expecting that ionizing radiation exposure increases CLL risk. The clinical characteristics of CLL, including prolonged latency and morbidity periods and a low case fatality rate, make it relatively difficult to evaluate associations between ionizing radiation and CLL risk via epidemiologic methods. The epidemiologic evidence of association between external exposure to ionizing radiation and CLL is weak. However, epidemiologic findings are consistent with a hypothesis of elevated CLL mortality risk after a latency and morbidity period that spans several decades. Our findings in this review suggest that there is not a persuasive basis for the conclusion that CLL is a nonradiogenic form of cancer.

Less than 5 years after the atomic bombings of Hiroshima and Nagasaki, it was established that there was an excess of leukemia among the atomic bomb survivors (Committee for the Compilation of Materials 1981). Japanese physicians noted the unusual number of leukemia cases among survivors, and researchers associated with the Atomic Bomb Casualty Commission (ABCC) subsequently confirmed the observation in a series of epidemiologic surveys (Folley et al. 1952; Valentine 1951). When examined by leukemia subtype, researchers with the ABCC reported substantial excesses of acute forms of leukemia and chronic myeloid leukemia among A-bomb survivors. In contrast, no excess of chronic lymphocytic leukemia (CLL) was observed (Finch et al. 1969; Ishimaru et al. 1969).

A few years later, Court-Brown and Doll (1957) reported the results of a study of mortality among adult British males who had received X-ray therapy for an arthritic condition (ankylosing spondylitis). When examining leukemia by subtype, it was noted that in the first 5 years postirradiation, deaths due to acute forms of leukemia and chronic myeloid leukemia were in substantial excess among these patients. The researchers found no excess of CLL (Court-Brown and Doll 1965; Darby et al. 1987). These findings, and their consistency with those of the A-bomb survivor studies, led investigators to postulate that there were differences in the radiogenicity of leukemia by subtype, with CLL being much less readily inducible by exposure to ionizing radiation than other types of leukemia (Darby et al. 1987).

Over time, this hypothesis has come to be expressed more strongly (Department of Health and Human Services 2002). Although most lymphatic and hematopoietic tissues are considered to be extremely sensitive to the carcinogenic effects of ionizing radiation, it is routinely presumed that CLL incidence is entirely insensitive to the carcinogenic effects of radiation. This assertion has become institutionalized in the U.S. Energy Employees Occupational Illness Compensation Program, under which all claims for CLL must be rejected because of the presumption that the risk of radiation-induced CLL is zero (Department of Health and Human Services 2002). In this article, we review the basis for the current presumption that CLL incidence is entirely unaffected by ionizing radiation exposure.

## Methods

In this article we present a review of the molecular, clinical, and epidemiologic evidence regarding the radiogenicity of CLL. We begin with a review of the current understanding of the molecular basis of CLL. Next, we review the clinical attributes of CLL and discuss the implications for etiologic research. Finally, we consider the epidemiologic literature on associations between external exposure to ionizing radiation and CLL risk. We focus on studies that have played a prominent role in the literature on the induction of leukemia, and specifically CLL, by ionizing radiation [National Research Council, Committee on the Biological Effects of Ionizing Radiation (BEIR V) 1990; United Nations Scientific Committee on the Effects of Atomic Radiation 2000]. Studies of the effects of exposure to ionizing radiation *in utero* or in childhood (e.g., for thymic enlargement or tinea capitis) were not included in this review because the average age of study participants at the end of follow-up tended to be less than the age at which CLL typically occurs.

The Revised European American Lymphoma classification scheme (Harris et al. 1994), which is widely accepted and was adopted by the World Health Organization, considers B-cell CLL and small lymphocytic lymphoma [SLL, a subtype of non-Hodgkin’s lymphoma (NHL)] to be a single disease entity, in recognition of the biologic and clinical similarities between these B-lymphocyte malignancies (Harris et al. 1999). Epidemiologic evidence of associations between ionizing radiation and risk of SLL would therefore be of interest in the context of this evaluation. However, epidemiologic studies have only recently begun to evaluate risk factors for SLL, and studies available for this review did not report results specifically for SLL.

Many of the epidemiologic studies that we reviewed reported results of analyses of standardized mortality ratios (SMRs) or standardized incidence ratios (SIRs). We have included 95% confidence intervals (CIs) for these findings. If a 95% CI was not reported in the text, we have calculated approximate 95% CIs (Rothman and Boice 1979). In this article we generically refer to measures of association based on odds ratios and rate ratios as estimates of relative risk (RR). Many of the studies that we reviewed reported estimates of radiation dose to the bone marrow. We have included these values in the text in order to allow comparison of the magnitude of doses between study populations. We report radiation dose estimates in millisieverts. Some of the reviewed papers reported dose estimates in milligrays, a physical quantity describing energy deposited per unit mass. For X-rays and gamma-rays, we assume a quality factor of unity; hence, 1 mGy = 1 mSv.

## Results

### Molecular basis of CLL.

CLL is a monoclonal disease of lymphocytes. Like other lymphoid cancers, CLL pathogenesis appears to be driven both by functional aberrations in immune function (Stevenson et al. 1998) and by somatic mutations, some of which may be a consequence of environmental exposures (Magrath 1992). Using contemporary molecular cytogenetic methods, chromosomal abnormalities are detected in most (> 80%) CLL cases (Stilgenbauer et al. 2002). Two tumor suppressor genes that are inactivated as a result of common CLL mutations, *p53* and *ATM*, are established causal contributors to malignant transformation. Therefore, these and other common somatic mutations are believed to play a causal role in the etiology of CLL.

The type of mutations observed in clonal cells obtained from CLL patients, primarily deletions of chromosomal material, require double-strand breaks of the chromosomal DNA in order to occur (Dewald et al. 2003; Stilgenbauer et al. 2000). Double-strand breaks (in one-half of the gene pair) are routinely generated during immunoglobulin gene rearrangement during normal lymphocyte maturation. However, in marked contrast with other lymphocytic malignancies, somatic mutations (specifically, translocations) involving immunoglobulin genes are rare in CLL (Stilgenbauer et al. 2002). This suggests that environmental exposures, rather than endogenous processes related to gene rearrangement during normal lymphocyte maturation, play an important role in producing the somatic mutations that contribute to the genesis of CLL.

It is well established that ionizing radiation has the ability to produce double-strand breaks in chromosomal DNA (United Nations Scientific Committee on the Effects of Atomic Radiation 2000). The primary mechanism by which biologic damage occurs is believed to be via the creation of ionized atoms and molecules that become chemically reactive. This can occur directly via ionization of a critical molecule, such as DNA, or indirectly via ionization of nearby molecules, such as water.

Like all cancers, CLL requires multiple mutations before neoplastic transformation occurs. Rather than arising due to a loss of cellular control and rapid proliferation of clonal lymphocytes, in the case of CLL the carcinogenic process is believed to typically involve an early event that causes a failure of apoptosis. The effectively immortalized lymphocyte may then persist in the body for years, increasing the likelihood that the cell will acquire additional mutations leading to full neoplastic transformation and the accumulation of clonal cells that may result in clinical symptoms of malignant disease (Voutsadakis 2000). This multistage process of neoplastic transformation is believed to account for many of the observed characteristics of the natural history of the disease and, specifically, the protracted induction and/or latency period associated with CLL.

Ionizing radiation exposure could play a role in one or more stages of this multistage process of neoplastic transformation. In addition, it is plausible that some early-stage mutational events may increase the likelihood of ionizing radiation exposure influencing later-stage transformations. For example, in a considerable proportion of CLL clones (~ 20%), the *ATM* gene is mutated; the *ATM* gene product is known to be involved in the repair of DNA double-strand breaks (Dunst et al. 1998; Humphreys et al. 1989; Jones et al. 1995; Parshad et al. 1985; Stilgenbauer et al. 2000), and mutations of this gene are associated with increased vulnerability to the carcinogenic effects of ionizing radiation. The inactivation of this tumor suppressor gene is not necessarily due to radiation exposure itself, but could occur as an early event that increases susceptibility to subsequent ionizing-radiation–induced mutations that result in progression of the carcinogenic process to CLL.

### Clinical aspects of CLL.

Compared with acute forms of leukemia and chronic myeloid leukemia, CLL is neither fast progressing nor highly fatal. With an increasing number of lymphocytes in the circulating bloodstream, eventually a person with CLL may present with symptoms of clinical significance such as shortness of breath, weight loss, or fever. However, often a patient with CLL is diagnosed during a routine medical examination that is not conducted because of any overt symptoms of disease (Rozman and Montserrat 1995).

The clinical aspects of the disease are important considerations from an epidemiologic perspective because they render CLL more prone to misclassification than acute lymphocytic and myeloid forms of leukemia. Even when patients present with overt symptoms of disease, diagnostic classification of CLL has long been characterized by a lack of consistency. Until recently, the same patient might be diagnosed with CLL by one hematologist and diagnosed with NHL by another, depending upon the classification scheme used by the diagnosing physician (Harris et al. 2000a, 2000b). CLL is now considered analogous to SLL (a subtype of NHL), the difference between the two being a function of the extent to which the tumor involves the bone marrow (CLL) versus solid tissue (SLL), with the recognition that both solid and circulating phases are present in many lymphoid neoplasms (Harris et al. 1999).

Particularly problematic are studies that rely on the use of cause of death information obtained from the death certificate as a proxy for information on CLL incidence. Cause of death information provides a relatively good measure of disease incidence if the disease progresses rapidly and has a high probability of leading to death. These are not the characteristics of CLL. Patients diagnosed with CLL often live many years without developing evidence of significant symptoms, and as a consequence of the typically old age at onset of CLL, many patients die with the disease, but from causes other than CLL (Crespo et al. 2003). In the United States, for example, the 5-year survival rate after a diagnosis of CLL is > 70% (Ries et al. 2003). Therefore, CLL is not necessarily the underlying cause of death recorded on a death certificate and, in fact, may not be indicated on the death certificate at all. In addition, deaths attributed to CLL tend to occur at very old ages when the validity of death certificate information tends to be poorest (Ron et al. 1994b). Furthermore, the direct repercussions or complications of CLL are often nonspecific, including immunodeficiency, and may increase the likelihood of infectious or malignant disease, thereby increasing the opportunity for conditions other than CLL to be recorded as the underlying cause of death. Secondary cancers frequently follow CLL incidence, and there is the possibility that the malignant clone of CLL can increase in malignancy due to additional chromosome breaks (dedifferentiation) and develop into a highly malignant B-cell NHL. Again, with high probability, the secondary cancer would be documented as the cause of death. This observation is supported by evidence from a recent study of patients with CLL in which Kyasa et al. (2004) found that the second malignancy was the primary cause of death recorded for 34% of CLL patient deaths.

### Epidemiologic findings regarding radiogenicity.

Table 1 lists a number of epidemiologic studies of populations exposed to ionizing radiation and describes the numbers of cases and study findings for associations with CLL by exposure type.

#### Atomic bomb.

The Lifespan Study (LSS) of the Japanese atomic bomb survivors has served as one of the primary studies for evaluation of the carcinogenic effects of ionizing radiation. However, for evaluation of CLL risk after exposure to ionizing radiation, the LSS provides minimal information because the incidence of CLL is extremely low in Asian populations (Finch and Linet 1992; Groves et al. 1995). Furthermore, much of the research published over the past 50 years on the effects of the atomic bomb on CLL incidence and mortality in the LSS suffered problems of case misclassification (Preston et al. 1994). After an extensive review of hematologic specimens for leukemia cases identified during the period from 1945 through 1980, it was determined that 7 of the 10 CLL cases registered during that period were, in fact, not CLL. These were determined to be cases of acute T-cell leukemia (ATL), a relatively common disease among Nagasaki residents regardless of their status as an A-bomb survivor. ATL is strongly related to infection by the human T-lymphotropic virus type 1, which is particularly prevalent in the Nagasaki region. Consequently, reports on radiation–CLL associations that are based on information collected before this reclassification of leukemia are of questionable reliability because of these problems of case misclassification. With the reclassification of leukemia cases, analyses of cancer incidence among 86,293 survivors (average bone marrow dose estimated as 300 mSv) over the period 1950–1987 include only four CLL cases. Given the small number of CLL cases, specific analyses of radiation–CLL associations have not been reported (Preston et al. 1994; Tomonaga et al. 1991).

#### Radiation therapy for nonmalignant disease.

Studies of patients treated by radiotherapy provide more informative results because they include larger numbers of CLL cases. Of particular importance, given the size of the study cohort, duration of follow-up, and average magnitude of radiation dose, are the results of a study of cancer mortality among approximately 14,000 British ankylosing spondylitis patients who were treated by X irradiation between 1935 and 1954 (average bone marrow dose estimated as 4,400 mSv). With vital status follow-up through 1991, it was found that these patients were more likely to have a death attributed to CLL than were members of the general population (observed = 7; SMR = 1.44; 95% CI, 0.6–2.8) (Weiss et al. 1995). Furthermore, consistent with expectations of long latency and morbidity periods for CLL mortality, excess CLL mortality was observed almost exclusively in the period ≥25 years after irradiation (in contrast to acute and myeloid leukemia, for which a peak in excess mortality was observed in the first 5 years posttreatment). Under a 25-year exposure lag assumption, a 2-fold excess of CLL mortality was observed (observed = 6; SMR = 1.97; 95% CI, 0.7–4.3) (Weiss et al. 1994).

Damber et al. (1995) examined the incidence of CLL in a cohort of 20,204 Swedish patients who were treated by radiotherapy between 1950 and 1964 for benign diseases of the locomotor system such as ankylosing spondylitis, arthrosis, and spondylosis (average bone marrow dose estimated as 400 mSv). Compared with the British ankylosing spondylitis patients, the radiation doses delivered to these patients were typically an order of magnitude lower, and only small parts of the body were irradiated (Damber et al. 1995). Patients were classified into three groups based on estimated radiation doses (< 0.20, 0.20–0.50, and > 0.50 Gy), and SIRs were calculated under a 0-year exposure lag assumption (there was no evaluation of variation in cancer risk with time since irradiation). There was a slight deficit of CLL among patients who received the lowest radiation doses (observed = 1; SIR = 0.94; 95% CI, 0.6–1.5) and a small excess of CLL among patients in the upper two dose groups (0.20–0.50 Gy: observed = 15; SIR = 1.17; 95% CI, 0.7–1.9; > 0.50 Gy: observed = 16; SIR = 1.18; 95% CI, 0.7–1.9).

Among 12,955 female patients who were treated by radiotherapy for benign gynecologic disorders (median dose to active bone marrow estimated as 1,200 mSv), CLL mortality rates (pooled together with lymphatic leukemia not otherwise specified) were elevated when compared with general population mortality rates (observed = 17; SMR = 1.8; 95% CI, 1.0–2.9) (Inskip et al. 1993). Consistent with expectations of a protracted latency and morbidity period, there was no excess of CLL mortality in the first 10 years of follow-up (observed = 1; SMR = 0.93; 95% CI, 0.0–5.2). In subsequent decades after irradiation, however, there was an excess of CLL mortality among irradiated patients. Under 20- and 30-year exposure lag assumptions, the ratios of observed to expected CLL deaths were 1.64 (observed = 10; 95% CI, 0.8–3.0) and 2.2 (observed = 7; 95% CI, 0.9–4.5), respectively. A comparison was also drawn using an internal referent population (a group of 3,185 patients with treatments other than radiotherapy). Comparisons between irradiated and nonirradiated patients by leukemia subtype produced highly unstable results because of the small number of leukemia cases in the nonirradiated group. The overall rate ratio for CLL comparing irradiated to nonirradiated patients was 1.1 (90% CI, 0.5–3.0); under 20- and 30-year exposure lag assumptions, the rate ratios for CLL comparing irradiated to nonirradiated patients were 1.3 and 2.3, respectively.

Investigations of CLL mortality among women treated by radiotherapy for excessive uterine bleeding (metropathia hemorrhagica) (Darby et al. 1994) and women treated by radiotherapy for infertility or amenorrhea (Ron et al. 1994a) have not reported on the risk of CLL after irradiation because of the small numbers of CLL cases (one and two CLL deaths, respectively) observed in these cohorts.

#### Radiotherapy for malignant disease.

Studies of cancer after radiotherapy treatment for a previous cancer offer the opportunity to study populations that have received relatively high doses of radiation. However, the radiation doses delivered for cancer therapy tend to be extremely high and localized; the intended effect is killing cells in the irradiated area that effectively prevents cancer induction. Although cell killing is also an issue in radiotherapy for benign diagnoses, in tumor irradiation it represents the original goal of the therapy, and attenuation of the dose–response relation for cancer induction may therefore be accentuated. In addition, the effects of radiation exposure on cancer incidence may differ for a healthy population than for a group of patients who are hospitalized for cancer treatment. Not only are these patients being treated for an existing cancer, but they also many receive chemotherapy in conjunction with radiotherapy, which may influence subsequent cancer incidence. Further, CLL tends to be a chronic disease with a prolonged latency period, and therefore survivorship is important for a diagnosis of CLL; if mortality rates for causes other than CLL differ with respect to radiotherapy, then bias may occur in estimates of radiation–CLL associations.

In a cohort study of second cancers after radiotherapy for invasive cancer of the uterine cervix among 182,040 women (average bone marrow dose was estimated as 7,100 mSv), Boice et al. (1985) examined the observed and expected (O/E) numbers of second cancers. In the first decade after irradiation, there were fewer than expected cases of CLL (observed = 9; O/E = 0.7; 95% CI, 0.3–1.3), whereas under a 20-year exposure lag assumption a small excess of CLL mortality was reported (observed = 3; O/E = 1.25; 95% CI, 0.3–3.7). A case–control study of secondary cancers after radiotherapy for invasive cancer of the uterine cervix was conducted building upon this cohort analysis (Boice et al. 1987). The study included leukemia cases that were diagnosed at least 1 year after diagnosis of cervical cancer, with four controls matched to each case. CLL incidence among patients treated by radiotherapy was compared with CLL incidence among patients treated by other means. No excess of CLL was observed when comparing patients treated by radiotherapy with other patients (RR = 1.03; 90% CI, 0.3–3.9). As indicated by the 90% CIs, the findings of the study are highly imprecise, largely because almost all cervical cancer cases were treated by radio-therapy, the treatment of choice during the study period. All results for analyses of CLL pertain to a 1-year exposure lag assumption with no evaluation of variation in risk with time since irradiation.

In case–control studies of leukemia after radiotherapy for invasive cancer of the uterine corpus (Curtis et al. 1994) and breast cancer (Curtis et al. 1989), leukemia cases were identified between 1935 and 1985 using cancer registry data, and controls were matched by cancer registry, age, year of diagnosis, and race. Among patients treated for cancer of the uterine corpus, the RR for CLL, comparing patients treated by radiotherapy with others, was 0.90 (95% CI, 0.4–1.9). Among patients treated by radiotherapy for breast cancer, the RR for CLL, comparing patients treated by radiotherapy with others, was 1.84 (95% CI, 0.5–6.7). Neither of these studies reported on evaluation of variation in the association between CLL and radiotherapy treatment with time since treatment.

#### Other studies of populations externally exposed to ionizing radiation.

The epidemiologic literature on cancer mortality among workers in the nuclear industry provides minimal basis for evaluating the effects of external exposure to ionizing radiation on CLL because of low statistical power. In analyses that combined mortality information on 95,673 nuclear industry workers in the United States, United Kingdom, and Canada (average cumulative dose was 40 mSv), a negative association between ionizing radiation exposure and CLL mortality was observed (excess RR per Sv = –0.95; 90% CI, –4.0 to 9.4). However, it stretches the practical limits of epidemiology to expect to directly estimate risk from occupational cohort data in which few cases are observed in the higher (e.g., ≥100 mSv ) dose range; of the 27 CLL cases observed in the international collaborative study of nuclear workers, only 1 case was observed among workers who had ≥100 mSv cumulative dose (Cardis et al. 1995). Furthermore, the reported results pertain to analyses under a 2-year exposure lag assumption. Under a reasonable exposure lag assumption for a slow-progressing disease like CLL (e.g., 20 years), the distribution of CLL cases with respect to cumulative radiation dose would tend to shift farther toward zero. Such considerations underline the limited power of nuclear worker cohort studies to derive radiation risk estimates for CLL mortality.

Studies of patients exposed to ionizing radiation via diagnostic X-ray procedures also provide minimal information about the association between ionizing radiation exposure and CLL incidence. For example, although cancer mortality has been examined among Massachusetts tuberculosis patients who were examined by X-ray fluoroscopy, no case of CLL was observed among these patients (Davis et al. 1989).

## Discussion

As ionizing radiation is transmitted through the human body, energy is transferred to the surrounding tissue and can produce biologic damage, including double-strand breaks in chromosomal DNA (United Nations Scientific Committee on the Effects of Atomic Radiation 2000). CLL appears to be similar to other hematologic malignancies whose pathogenesis involves structural changes on the chromosomal level that cause mutational changes on the molecular level, altering important cellular functions, and, ultimately, leading to malignant transformation of a cell (Irons and Stillman 1996). Therefore, at the level of DNA damage, there is no basis for the assumption that the association between ionizing radiation exposure and CLL risk would be zero. Rather, there is strong evidence that the somatic mutations that contribute to the genesis of CLL (in a process that is likely to also involve aberrations in immune function) can be produced by ionizing radiation exposure.

Given the radiobiologic plausibility of radiation-induced CLL, one would expect that the conclusion that CLL is nonradiogenic would be supported by a strong, consistent body of epidemiologic evidence indicating that CLL is an exception to the general principles of radiation carcinogenesis. This is not the case. Rather, there is limited epidemiologic evidence with which to evaluate the relative radiogenicity of CLL. Most studies include small numbers of cases, and few have conducted analyses to adequately account for the prolonged latency and morbidity periods of CLL.

A simple and parsimonious alternative to the hypothesis that CLL is entirely insensitive to ionizing radiation effects is that radiation does influence CLL incidence, but this association is more difficult to identify via epidemiologic methods than the association between ionizing radiation and acute lymphocytic and myeloid forms of leukemia. Acute lymphocytic and myeloid forms of leukemia arise as a consequence of an increased rate of mitosis (due to loss of cellular control over proliferation of transformed cells). Consequently, the number of white blood cells in the bone marrow and/or circulating in the bloodstream of a patient with acute lymphocytic or myeloid leukemia may increase dramatically over a relatively short period of time. In contrast, the fundamental mechanism of accumulation of CLL-clonal cells is an extension of the life span of the transformed lymphocytes due to a failure of apoptosis (Voutsadakis 2000), which leads to a gradual accumulation of circulating CLL cells. Thus, although clinical symptoms such as shortness of breath, weight loss, or fever may slowly develop over time, CLL is often diagnosed during routine physical examination of asymptomatic elderly patients.

This long asymptomatic period (followed by a protracted period of morbidity) has important implications for epidemiologic investigations of radiation–CLL associations. It means that case ascertainment may be poor and partly obscured by competing causes of death. Analytically, in order for an investigation of radiation-induced CLL mortality to detect an effect, the study must encompass a period of follow-up that is long enough to allow for an extended induction, latency, and morbidity period after exposure occurs. Studies with short duration of follow-up (e.g., one or two decades) could observe no effect of ionizing radiation on CLL simply because the time from exposure to end of follow-up is less than the minimal induction, latency, and morbidity period for radiation-induced CLL mortality. Furthermore, the ability to detect an association, if one exists, requires relating CLL incidence or mortality to exposures in the distant past using appropriate methods of survival analysis. If the effect of radiation on CLL risk only becomes apparent many years (or a few decades) after irradiation, then analyses conducted under relatively short exposure lag assumptions may suffer serious exposure misclassification problems.

The Revised European American Lymphoma classification scheme (Harris et al. 1994) reflects a recent attempt to use immunophenotypic and genetic characteristics in order to classify lymphomas into subgroups that share common clinical and pathologic characteristics. To the extent that refinements in disease classification improve the ability to identify cancer cases that are similar in terms of etiology and natural history (e.g., durations of latency and morbidity periods), these efforts should strengthen epidemiologic investigations. However, constructing nosologic schemes primarily with reference to considerations about disease management and prognosis rather than etiology, and classifying diseases into increasingly refined categories, poses potential problems for epidemiologic research. Evidence of the hazardous effects of an exposure may be obscured by classification of exposure-induced cases into different groups based upon clinical characteristics that are not etiologically relevant. Further, as the classification of diseases becomes refined, it becomes increasingly difficult to conduct statistical analyses with adequate power to address questions about the effects of an exposure on disease incidence. Given the ability to construct ever more refined disease categorizations, it may be increasingly important to identify mechanistic and etiologic grounds for aggregation of subtypes of diseases for epidemiologic research purposes.

## Conclusion

The assumption, under existing federal regulations, that the risk of CLL after exposure to ionizing radiation is zero is unlikely to be correct. In order to be correct, CLL must be an exception to general principles of radiation carcinogenesis. In this review we found no support for that conclusion. Current understanding of the pathogenesis of CLL describes a process in which there is an important role played by mutational events that can be produced by exposure to ionizing radiation. The epidemiologic evidence of radiation–CLL associations is weak; however, given the limitations of the reviewed studies, these findings do not offer a persuasive basis for concluding that CLL is an exception to general principles of radiation carcinogenesis. In addition, there is a problem of logical inconsistency if the government continues to assert that CLL is nonradiogenic whereas SLL is radiogenic. Contemporary classification schemes hold that B-cell CLL and SLL are analogous diseases and should be considered as a single disease entity. It is possible that the magnitude of the association between ionizing radiation and CLL is smaller than that for other lymphomas and leukemias; evaluation of the magnitude of this association is difficult given the limitations of existing epidemiologic data. Nonetheless, it is likely that CLL incidence, like other forms of cancer, will be increased by exposure to ionizing radiation.

## Figures and Tables

**Table 1:** Epidemiologic studies of populations exposed to ionizing radiation and risk of CLL: numbers of cases and summary of study findings by type of exposure.

Type of exposure/study	Reference	CLL cases	Radiation risk
Atomic bomb			
Japanese survivors	Preston et al. 1994	4	NR
Radiotherapy			
Ankylosing spondylitis	Weiss et al. 1994, 1995	7	+*^a^*
Benign disorders of the locomotor system	Damber et al. 1995	17	+*^b^*
Benign gynecologic disorders	Inskip et al. 1993	17	+*^a^*
Cervical cancer	Boice et al. 1985, 1987	52	−
Uterine cancer	Curtis et al. 1994	54	−
Breast cancer	Curtis et al. 1989	10	+
Occupation			
Nuclear industry	Cardis et al. 1995	27	−

Abbreviations: –, no evidence of radiation risk; +, evidence for radiation risk; NR, results not reported.

a For the period ≥25 years after irradiation.

b Among those receiving ≥0.20 Gy.

## References

[b1-ehp0113-000001] Boice JD, Blettner M, Kleinerman RA, Stovall M, Moloney WC, Engholm G (1987). Radiation dose and leukemia risk in patients treated for cancer of the cervix. J Natl Cancer Inst.

[b2-ehp0113-000001] Boice JD, Day NE, Andersen A, Brinton LA, Brown R, Choi NW (1985). Second cancers following radiation treatment for cervical cancer. An international collaboration among cancer registries. J Natl Cancer Inst.

[b3-ehp0113-000001] Cardis E Gilbert E Carpenter L Howe G Kato I Fix J 1995. Combined Analyses of Cancer Mortality Among Nuclear Industry Workers in Canada, the United Kingdom and the United States of America. IARC Technical Report No. 25. Lyon, France:International Agency for Research on Cancer.

[b4-ehp0113-000001] Committee for the Compilation of Materials on Damage Caused by the Atomic Bombs in Hiroshima and Nagasaki 1981. Hirosima and Nagasaki: The Physical, Medical, and Social Effects of the Atomic Bombings. Tokyo:Iwanami Shoten.

[b5-ehp0113-000001] Court-Brown W Doll R 1957. Leukemia and Aplastic Anaemia in Patients Irradiated for Anklyosing Spondylitis. Report no. 295. London:Her Majesty’s Stationery Office.

[b6-ehp0113-000001] Court-Brown W, Doll R (1965). Mortality from cancer and other causes after radiotherapy for ankylosing spondylitis. Br Med J.

[b7-ehp0113-000001] Crespo M, Bosch F, Villamor N, Bellosillo B, Colomer D, Rozman M (2003). ZAP-70 expression as a surrogate for immunoglobulin-variable-region mutations in chronic lymphocytic leukemia. N Engl J Med.

[b8-ehp0113-000001] Curtis RE, Boice JD, Stovall M, Bernstein L, Holowaty E, Karjalainen S (1994). Relationship of leukemia risk to radiation dose following cancer of the uterine corpus. J Natl Cancer Inst.

[b9-ehp0113-000001] Curtis RE, Boice JD, Stovall M, Flannery JT, Moloney WC (1989). Leukemia risk following radiotherapy for breast cancer. J Clin Oncol.

[b10-ehp0113-000001] Damber L, Larsson LG, Johansson L, Norin T (1995). A cohort study with regard to the risk of haematological malignancies in patients treated with X-rays for benign lesions in the locomotor system. I. Epidemiological analyses. Acta Oncol.

[b11-ehp0113-000001] Darby SC, Doll R, Gill SK, Smith PG (1987). Long term mortality after a single treatment course with X-rays in patients treated for ankylosing spondylitis. Br J Cancer.

[b12-ehp0113-000001] Darby SC, Reeves G, Key T, Doll R, Stovall M (1994). Mortality in a cohort of women given X-ray therapy for metropathia haemorrhagica. Int J Cancer.

[b13-ehp0113-000001] Davis FG, Boice JD, Hrubec Z, Monson RR (1989). Cancer mortality in a radiation-exposed cohort of Massachusetts tuberculosis patients. Cancer Res.

[b14-ehp0113-000001] Department of Health and Human Services (2002). 42 CFR Parts 81 and 82. Guidelines for Determining the Probability of Causation and Methods for Radiation Dose Reconstruction Under the Employees Occupational Illness Compensation Program Act of 2000; Final Rules. Fed Reg.

[b15-ehp0113-000001] Dewald GW, Brockman SR, Paternoster SF, Bone ND, O’Fallon JR, Allmer C (2003). Chromosome anomalies detected by interphase fluorescence in situ hybridization: correlation with significant biological features of B-cell chronic lymphocytic leukaemia. Br J Haematol.

[b16-ehp0113-000001] Dunst J, Neubauer S, Becker A, Gebhart E (1998). Chromosomal in-vitro radiosensitivity of lymphocytes in radiotherapy patients and AT-homozygotes. Strahlenther Onkol.

[b17-ehp0113-000001] Finch SC, Hoshino T, Itoga T, Ichimaru M, Ingram RH (1969). Chronic lymphocytic leukemia in Hiroshima and Nagasaki, Japan. Blood.

[b18-ehp0113-000001] Finch SC, Linet MS (1992). Chronic leukaemias. Baillieres Clin Haematol.

[b19-ehp0113-000001] Folley J, Borges W, Yamawaki T (1952). Incidence of leukemia in survivors of the atomic bomb. Am J Med.

[b20-ehp0113-000001] Groves FD, Linet MS, Devesa SS (1995). Patterns of occurrence of the leukaemias. Eur J Cancer.

[b21-ehp0113-000001] Harris NL, Jaffe ES, Diebold J, Flandrin G, Muller-Hermelink HK, Vardiman J (1999). World Health Organization classification of neoplastic diseases of the hematopoietic and lymphoid tissues: report of the Clinical Advisory Committee meeting—Airlie House, Virginia, November 1997. J Clin Oncol.

[b22-ehp0113-000001] Harris NL, Jaffe ES, Diebold J, Flandrin G, Muller-Hermelink HK, Vardiman J (2000a). Lymphoma classification—from controversy to consensus: the R.E.A.L. and WHO Classification of lymphoid neoplasms. Ann Oncol.

[b23-ehp0113-000001] Harris NL, Jaffe ES, Diebold J, Flandrin G, Muller-Hermelink HK, Vardiman J (2000b). The World Health Organization classification of hematological malignancies report of the Clinical Advisory Committee meeting, Airlie House, Virginia, November 1997. Mod Pathol.

[b24-ehp0113-000001] Harris NL, Jaffe ES, Stein H, Banks PM, Chan JKC, Cleary ML (1994). A revised European-American classification of lymphoid neoplasms: a proposal from the International Lymphoma Study Group. Blood.

[b25-ehp0113-000001] Humphreys MW, Nevin NC, Wooldridge MA (1989). Cytogenetic investigations in a family with ataxia telangiectasia. Hum Genet.

[b26-ehp0113-000001] Inskip PD, Kleinerman RA, Stovall M, Cookfair DL, Hadjimichael O, Moloney WC (1993). Leukemia, lymphoma, and multiple myeloma after pelvic radiotherapy for benign disease. Radiat Res.

[b27-ehp0113-000001] Irons RD, Stillman WS (1996). The process of leukemogenesis. Environ Health Perspect.

[b28-ehp0113-000001] Ishimaru T Hoshino T Ichimaru M Okada H Tomiyasu T Tsuchimoto T 1969. Leukemia in Atomic Bomb Survivors: Hiroshima and Nagasaki. TR-25-69. Hiroshima, Japan:Atomic Bomb Casualty Commission.

[b29-ehp0113-000001] Jones LA, Scott D, Cowan R, Roberts SA (1995). Abnormal radiosensitivity of lymphocytes from breast cancer patients with excessive normal tissue damage after radiotherapy: chromosome aberrations after low dose-rate irradiation. Int J Radiat Biol.

[b30-ehp0113-000001] Kyasa MJ, Hazlett L, Parrish RS, Schichman SA, Zent CS (2004). Veterans with chronic lymphocytic leukemia/small lymphocytic lymphoma (CLL/SLL) have a markedly increased rate of second malignancy, which is the most common cause of death. Leuk Lymphoma.

[b31-ehp0113-000001] Magrath I (1992). Molecular basis of lymphomagenesis. Cancer Res.

[b32-ehp0113-000001] National Research Council, Committee on the Biological Effects of Ionizing Radiation (BEIR V) 1990. Health Effects of Exposure to Low Levels of Ionizing Radiation (BEIR V). Washington, DC:National Academy Press.

[b33-ehp0113-000001] Parshad R, Sanford KK, Jones GM, Tarone RE (1985). G2 chromosomal radiosensitivity of ataxia-telangiectasia heterozygotes. Cancer Genet Cytogenet.

[b34-ehp0113-000001] Preston DL, Kusumi S, Tomonaga M, Izumi S, Ron E, Kuramoto A (1994). Cancer incidence in atomic bomb survivors. Part III. Leukemia, lymphoma and multiple myeloma, 1950–1987. Radiat Res.

[b35-ehp0113-000001] Ries LAG Eisner MP Kosary CL Hankey BF Miller BA Clegg L eds. 2003. SEER Cancer Statistics Review, 1975–2000. Bethesda, MD:National Cancer Institute. Available: http://seer.cancer.gov/csr/1975_2000/ [accessed 18 November 2004].

[b36-ehp0113-000001] Ron E, Boice JD, Hamburger S, Stovall M (1994a). Mortality following radiation treatment for infertility of hormonal origin or amenorrhoea. Int J Epidemiol.

[b37-ehp0113-000001] Ron E, Carter R, Jablon S, Mabuchi K (1994b). Agreement between death certificate and autopsy diagnoses among atomic bomb survivors. Epidemiology.

[b38-ehp0113-000001] Rothman K Boice JD 1979. Epidemiologic Analyses with a Programmable Calculator. NIH Publication No. 79-1649. Washington, DC:U.S. Government Printing Office.

[b39-ehp0113-000001] Rozman C, Montserrat E (1995). Chronic lymphocytic leukemia. N Engl J Med.

[b40-ehp0113-000001] Stevenson F, Sahota S, Zhu D, Ottensmeier C, Chapman C, Oscier D (1998). Insight into the origin and clonal history of B-cell tumors as revealed by analysis of immunoglobulin variable region genes. Immunol Rev.

[b41-ehp0113-000001] Stilgenbauer S, Bullinger L, Lichter P, Dohner H (2002). Genetics of chronic lymphocytic leukemia: genomic aberrations and V(H) gene mutation status in pathogenesis and clinical course. Leukemia.

[b42-ehp0113-000001] Stilgenbauer S, Lichter P, Dohner H (2000). Genetic features of B-cell chronic lymphocytic leukemia. Rev Clin Exp Hematol.

[b43-ehp0113-000001] Tomonaga M Matsuo T Carter RL Bennett JM Kuriyama K Imanaka F 1991. Differential effects of atomic bomb irradiation in inducing major leukemia types: analyses of open-city cases including the life span study cohort based upon updated diagnostic systems and the dosimetry system 1986 (DS86). TR 9-91. Hiroshima, Japan:Radiation Effects Research Foundation.

[b44-ehp0113-000001] United Nations Scientific Committee on the Effects of Atomic Radiation 2000. Sources and Effects of Ionizing Radiation. Vol. II: Effects. New York:United Nations.

[b45-ehp0113-000001] Valentine W 1951. Present Status of the Study of the Incidence of Leukemia Among Individuals Surviving Exposure to the Atomic Bomb in Hiroshima and Nagasaki. Hiroshima, Japan: Atomic Bomb Casualty Commission.

[b46-ehp0113-000001] Voutsadakis IA (2000). Apoptosis and the pathogenesis of lymphoma. Acta Oncol.

[b47-ehp0113-000001] Weiss HA, Darby SC, Doll R (1994). Cancer mortality following X-ray treatment for ankylosing spondylitis. Int J Cancer.

[b48-ehp0113-000001] Weiss HA, Darby SC, Fearn T, Doll R (1995). Leukemia mortality after X-ray treatment for ankylosing spondylitis. Radiat Res.

